# The c-Myc/AKT1/TBX3 Axis Is Important to Target in the Treatment of Embryonal Rhabdomyosarcoma

**DOI:** 10.3390/cancers12020501

**Published:** 2020-02-21

**Authors:** Danica Sims, Hapiloe Mabaruti Maranyane, Victoria Damerell, Dhirendra Govender, Ashwin W. Isaacs, Jade Peres, Sharon Prince

**Affiliations:** 1 Division of Cell Biology, Department of Human Biology, Faculty of Health Sciences, University of Cape Town, Observatory, Cape Town 7925, South Africa; SMTDAN013@myuct.ac.za (D.S.); hapiloe.m@gmail.com (H.M.M.); victoria.damerell@gmx.de (V.D.); ash.isaacs@uct.ac.za (A.W.I.); jade.peres@uct.ac.za (J.P.); 2 Division of Anatomical Pathology, Department of Pathology, Faculty of Health Sciences, University of Cape Town, Observatory, Cape Town 7925, South Africa; dhiren.govender@uct.ac.za

**Keywords:** T-box transcription factor-3 (TBX3), rhabdomyosarcoma (RMS), embryonal rhabdomyosarcoma (ERMS), childhood cancer, AKT1, c-Myc, oncogene

## Abstract

Rhabdomyosarcoma is a highly aggressive malignant cancer that arises from skeletal muscle progenitor cells and is the third most common solid tumour in children. Despite significant advances, rhabdomyosarcoma still presents a therapeutic challenge, and while targeted therapy has shown promise, there are limited options because the molecular drivers of rhabdomyosarcoma are poorly understood. We previously reported that the T-box transcription factor 3 (TBX3), which has been identified as a druggable target in many cancers, is overexpressed in rhabdomyosarcoma patient samples and cell lines. To identify new molecular therapeutic targets to treat rhabdomyosarcoma, this study investigates the potential oncogenic role(s) for TBX3 and the factors responsible for upregulating it in this cancer. To this end, rhabdomyosarcoma cell culture models in which TBX3 was either stably knocked down or overexpressed were established and the impact on key hallmarks of cancer were examined using growth curves, soft agar and scratch motility assays, as well as tumour-forming ability in nude mice. Our data show that TBX3 promotes substrate-dependent and -independent proliferation, migration and tumour formation. We further reveal that TBX3 is upregulated by c-Myc transcriptionally and AKT1 post-translationally. This study identifies c-Myc/AKT1/TBX3 as an important axis that could be targeted for the treatment of rhabdomyosarcoma.

## 1. Introduction

Rhabdomyosarcoma (RMS), an aggressive and highly malignant cancer of the skeletal muscle, is the most common soft tissue sarcoma in children and adolescents and it is the third most prevalent extracranial solid childhood tumour after neuroblastoma and Wilms tumour [1,2,3]. Embryonal (ERMS) and alveolar (ARMS) are the two main subtypes of RMS, and whereas ERMS accounts for ~75% of RMS and occurs mostly in children, ARMS arises mostly in adolescents and young adults [2,4]. ERMS is characterized by genetic aberrations, such as loss of heterozygosity at chromosome 11p15, a region that harbours *insulin growth factor (IGF) 2*, or gains at chromosomes 2, 8, 12 and 13 [4,5,6,7]. Both RMS subtypes express myogenic regulatory factors, such as MyoD and myogenin, and it has thus been postulated that RMS arises from a myogenic precursor cell that failed to correctly and completely differentiate into muscle cells. However, RMS tumours can originate in non-muscle sites, and therefore their aetiology is under debate [8]. 

The current treatment modalities for RMS include a combination of surgery, radiation and chemotherapy, and while this has increased the 5-year overall survival rate for non-metastatic RMS to approximately 75%, outcomes for patients with metastatic RMS remain dismal. Furthermore, current therapies are associated with severe toxicity and debilitating side-effects, and approximately a third of patients with non-metastatic RMS on treatment continue to experience tumour growth and/or tumour recurrence [9]. While targeted therapies have dramatically advanced the treatment of numerous cancers, their application in RMS has been slow. Indeed, there are only a few available single-targeted therapies for RMS and they show efficacy in only a subset of patients and responses to them are short-lived due to treatment resistance [9,10,11]. A major challenge in the treatment of RMS is therefore to identify key molecular targets which will lead to the development of single and combination therapies. 

*T-box transcription factor 3 (TBX3)* is a member of the developmentally important T-box transcription factor family. Human *TBX3* consists of seven exons and alternative processing and splicing gives rise to two predominant isoforms, *TBX3* and *TBX3+2a*, with *TBX3* being the more dominant of the two. *TBX3+2a* results from alternative splicing of the second intron which leads to the addition of the +2a exon, and consequently this isoform has an additional 20 amino acids within the T-box DNA-binding domain [12,13]. While some studies have shown that TBX3 and TBX3+2a have similar roles, at least functionally if not always mechanistically, there is also evidence that their functions may vary across different cell types [12,13,14,15,16]. TBX3 is critical for the formation of, amongst other structures, the heart, mammary glands and the musculoskeletal system of the limbs, and, when mutated, causes the ulnar mammary syndrome [12,17,18]. In addition, TBX3 is overexpressed in a myriad of carcinomas and sarcomas, where it contributes to multiple aspects of the oncogenic process [13,19,20,21,22,23,24,25,26,27,28,29]. For example, TBX3 bypasses senescence by repressing *p14/p19^ARF^* in breast cancer and promotes proliferation by repressing *p21^WAFI/CIPI/SDII^* (referred to as *p21*) in chondrosarcoma, *p57^KIP2^* in papillary thyroid carcinoma, or *phosphatase and tensin homologue (PTEN)* in head and neck squamous cell carcinoma [24,28,30,31]. Furthermore, TBX3 promotes the migration of melanoma cells through directly repressing the cell adhesion protein, E-cadherin, and angiogenesis in pancreatic ductal adenocarcinomas which correlated with increased expression of *VEGF-A* and *FGF2* [32,33,34,35]. Recently, TBX3 was shown to be expressed in a panel of patient-derived RMS tissue sections and the transient knockdown of TBX3 significantly decreased ERMS cell migration [36]. However, whether TBX3 contributes to other aspects of rhabdomyosarcomagenesis and the mechanism(s) responsible for upregulating it in RMS is not known.

Several lines of evidence suggest that c-Myc, a basic region/helix–loop–helix/leucine zipper (b/HLH/Zip) transcription factor, is an important oncogenic signalling molecule in RMS [37]. Indeed, upregulated levels of c-Myc is often associated with tumour aggression and poor clinical outcome [38,39,40,41,42,43,44,45,46,47]. Furthermore, c-Myc was shown to function as a pro-proliferative and anti-apoptotic factor in RMS by repressing p21, and when it was depleted in ERMS cell lines a number of metastatic, invasive and angiogenic markers decreased [42,48,49]. In addition, c-Myc conferred radio-resistance by protecting ERMS cells from apoptosis and DNA damage and promoting DNA repair [48]. Interestingly, c-Myc transcriptionally activates TBX3 by directly binding two E-boxes, and this regulation was shown to be important for promoting chondrosarcoma cell proliferation [50]. 

Aberrant PI3K/AKT signalling has been described in many human cancers, including soft tissue sarcomas [51,52,53,54,55,56,57], and AKT activation contributes to pathways that promote tumour cell proliferation, invasion and metastasis [58,59]. Indeed, there is compelling evidence that a key requirement for the development of RMS is the prolonged activation of serine/threonine kinases such as AKT [60]. In addition, tissue microarray data have revealed that AKT is frequently phosphorylated and activated in ARMS and ERMS and this activation was negatively associated with patient survival [61,62]. Furthermore, RAS proteins are GTPases that function as molecular switches that control proliferation and cell survival, and ERMS is driven by mutations in RAS proteins which promote oncogenesis [63,64,65]. Moreover, RAS/MAPK signalling enhances MYC expression and stability and IGF2 was shown to be overexpressed in RMS and to drive AKT activation [66,67,68]. This highlights the involvement of a complex network of pathways which sustain the ERMS phenotype. There are three AKT isoforms (AKT1, AKT2 and AKT3) and they have very distinct roles in specific cell lineages with important consequences for cellular physiology [69,70]. Indeed, in melanoma, AKT3 is the most abundant isoform and AKT phosphorylation of TBX3 enhances protein stability, nuclear localisation and transcriptional activity [71]; in fibrosarcoma and chondrosarcoma, AKT1 is the predominant isoform and it maintains high levels of TBX3 [72]. 

With the aim of identifying novel therapeutic targets to treat RMS, we investigated whether TBX3 is a key oncoprotein downstream of c-Myc and AKT in RMS. We demonstrate that a c-Myc/AKT/TBX3-driven process promotes cell proliferation, anchorage-independent growth, cell migration, tumour formation and invasion of ERMS cells. Therapeutic strategies aimed at targeting this novel c-Myc/AKT/TBX3 pathway may significantly improve the survival of ERMS patients. 

## 2. Results

### 2.1. TBX3 Is Overexpressed in ERMS

**Figure 1:**
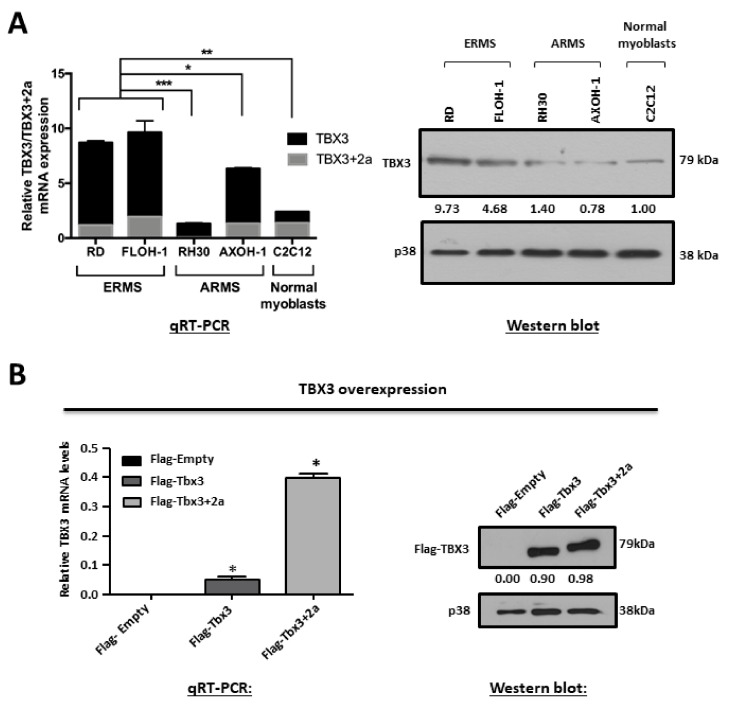

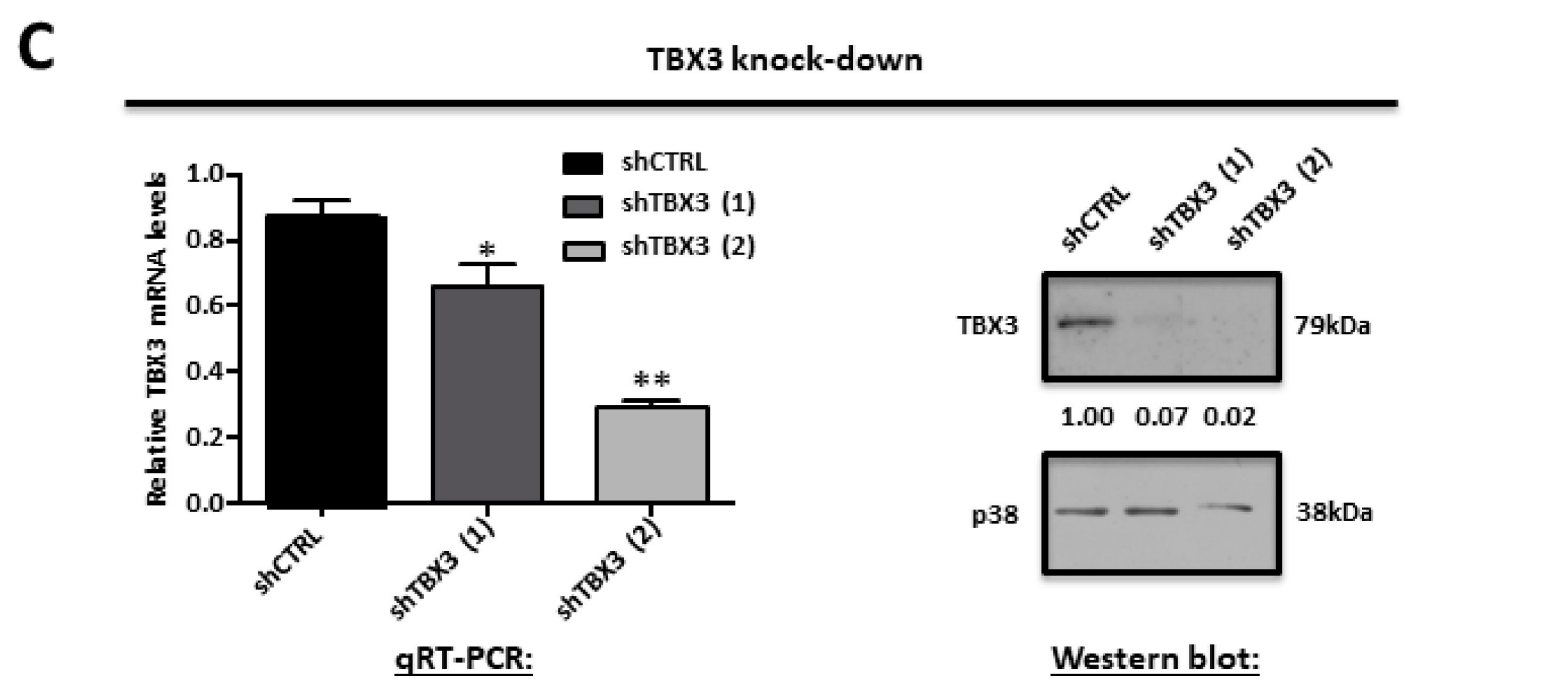
TBX3 is overexpressed in embryonal rhabdomyosarcoma (ERMS). (**A**) Total RNA and protein were extracted from RMS cell lines (ERMS: RD, FLOH-1 and ARMS: RH30 and AXOH-1) and normal muscle myoblasts (C2C12). (Left) qRT-PCR was performed on reverse transcribed RNA using primers specific to TBX3, TBX3+2a and GUSB/GusB (housekeeping gene) and mRNA levels were normalised to GUSB/GusB and quantified relative to C2C12. (Right) protein extracts were subjected to Western blot analysis with antibodies specific to TBX3 and p38 (loading control). (**B**,**C**) Establishment of ERMS cell culture models in which TBX3 was either stably overexpressed or knocked down in the RD cell line. mRNA and protein from (**B**) RD Flag-Empty, Flag-Tbx3 or Flag-Tbx3+2a cell lines and (**C**) RD shCTRL and shTBX3 clones were subjected to (left) qRT-PCR with primers specific to TBX3 and GUSB and mRNA levels were normalised to GUSB and (right) Western blotting with antibodies to (**B**) Flag-tag (representative of ectopically expressed Flag-TBX3 protein) and (**C**) TBX3 and p38 was used as a loading control. Densitometric readings for all Western blots were calculated relative to the p38 control. The values in the graphs represent the mean of at least three independent experiments ± SEM (* *p* < 0.02; ** *p* < 0.002; *** *p* < 0.0001).

To determine whether TBX3 overexpression is a common feature of RMS cells, the status of TBX3 was determined in ERMS (RD and FLOH-1) and ARMS (RH30 and AXOH-1) cell lines. The results from qRT-PCR analyses with primers to the TBX3 and TBX3+2a isoforms show that, compared to the C2C12 normal myoblasts, TBX3 mRNA levels were significantly higher in the ERMS cell lines (Figure 1A, left panel). Furthermore, the ERMS cell lines had higher levels of TBX3 mRNA and protein compared to the ARMS cell lines (Figure 1A). 

### 2.2. Establishment of ERMS Cell Culture Models in which TBX3 Was Either Stably Knocked Down or Overexpressed 

To determine the significance of TBX3 expression in ERMS, cell culture models were established in which TBX3 was either stably overexpressed or knocked down in RD cells, and we measured the impact of altering TBX3 levels on several hallmarks of cancer. The overexpression cell culture model was generated by the stable transfection of RD cells with Flag-Tbx3, Flag-Tbx3+2a or Flag-Empty. To generate the knock down model, RD cells were stably transfected with a pSuper.neo/GFP expression vector containing a short-hairpin (sh) RNA sequence that targets TBX3 (shTBX3) or a non-specific control sequence (shCTRL) [32]. G418-resistant clones were isolated and expanded into cell lines and Figure 1B,C show the positive clones that were selected for further analyses.

### 2.3. TBX3 Overexpression Promotes Proliferation, Anchorage Independent Growth and Migration of ERMS Cells 

**Figure 2:**
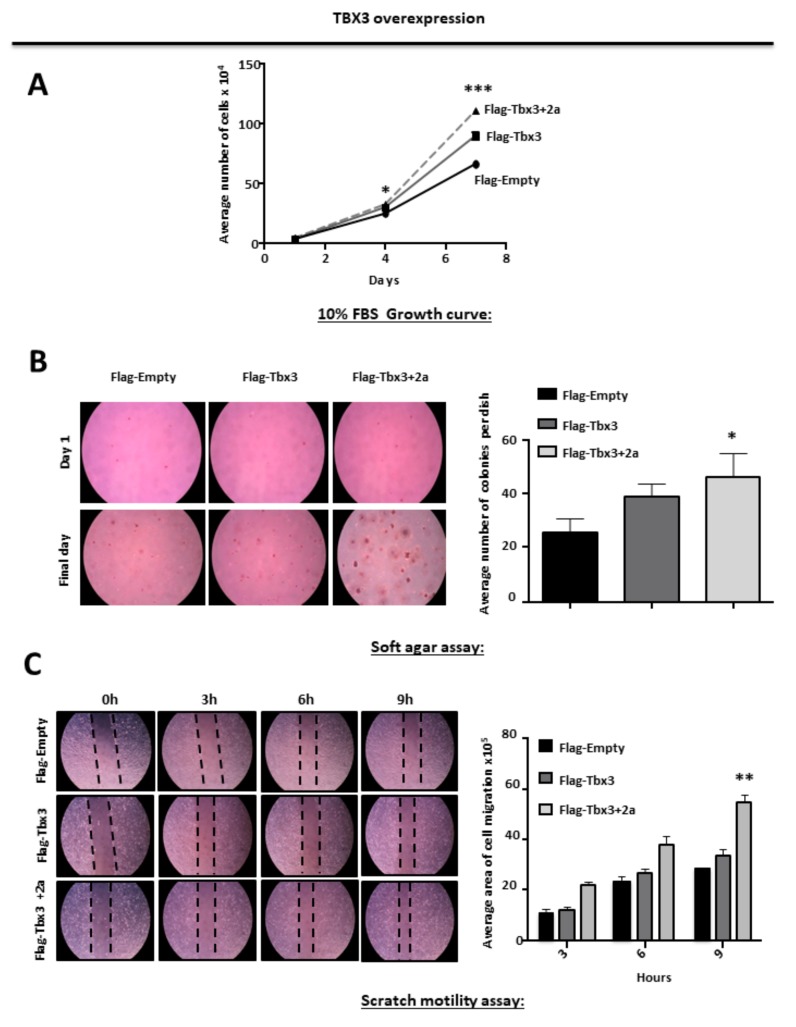
TBX3 overexpression promotes proliferation, anchorage-independent growth and migration of ERMS cells. (**A**) Growth curve analyses of ERMS (RD) Flag-Empty, Flag-Tbx3 and Flag-Tbx3+2a cell lines. (**B**) RD Flag-Empty, Flag-Tbx3 and Flag-Tbx3+2a cell lines were suspended in soft-agar medium slurry and allowed to proliferate for 14 days. Whole dishes were stained with p-iodinitrotetrazolium. (Left) Representative images are shown (200× magnification). (Right) Quantification of at least 40 fields of view from four independent experiments. (**C**) Cell motility assays were performed to measure the migration of RD Flag-Empty, Flag-Tbx3 and Flag-Tbx3+2a cells. A linear wound was made on confluent cells and distance migrated was measured at 3, 6 and 9 h. The values in the graphs represent the mean of at least four independent experiments ± SEM (* *p* < 0.05, ** *p* < 0.001, *** *p* < 0.0001).

Growth curves and soft agar assays were conducted to investigate the effect of ectopically overexpressing Tbx3 and Tbx3+2a isoforms on RD substrate-dependent and -independent cell proliferation, respectively. The results show that both Tbx3 isoforms promoted the proliferation of RD cells in the presence and absence of a substrate (Figure 2A,B). Furthermore, two-dimensional scratch motility assays show that overexpression of both Tbx3 isoforms increased RD cell motility (Figure 2C). It is important to note that the results obtained in the soft agar and scratch motility assays were not statistically significant for the cells expressing Flag-Tbx3.These results suggest that Tbx3+2a may be more efficient at promoting these hallmarks of cancer in ERMS. 

### 2.4. TBX3 Overexpression Promotes Tumour forming Ability and Invasiveness of ERMS Xenografts

**Figure 3:**
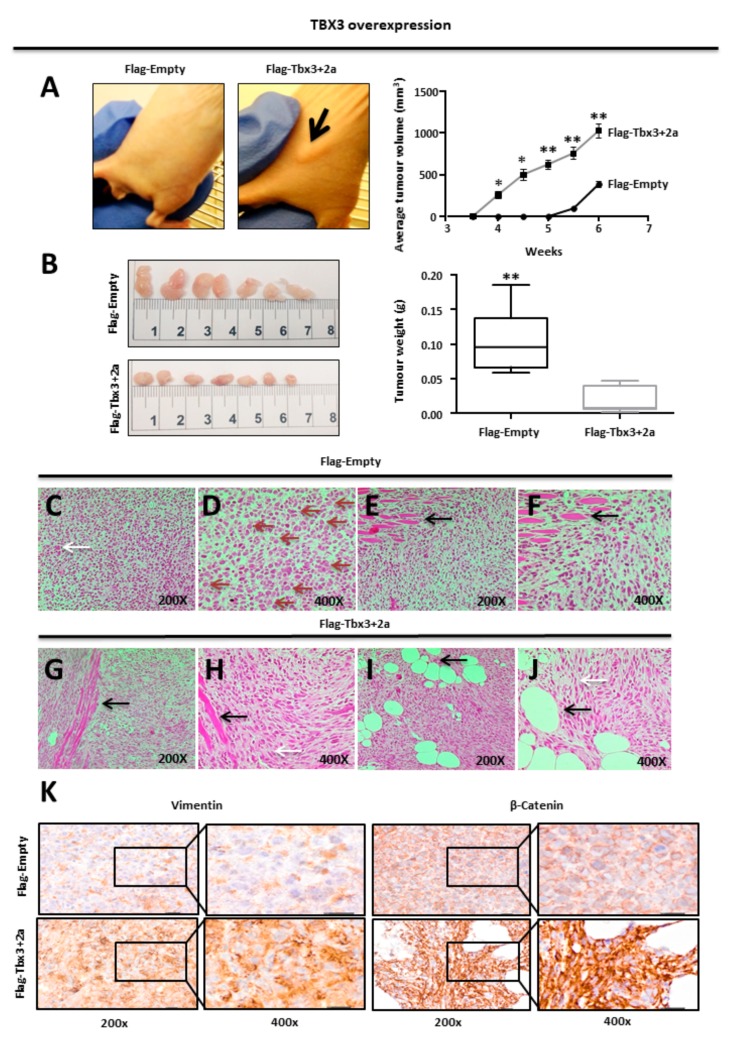
TBX3 overexpression promotes tumour-forming ability and invasiveness of ERMS xenografts. (**A**) RD Flag-Empty and Flag-Tbx3+2a cells were injected subcutaneously into the flanks of immunocompromised nude mice (N = 7 each). Mice were photographed at week 4 once visible tumour formation occurred (indicated by the black arrow) (top left panel). In situ tumour volume (mm^3^) was measured over 6 weeks using calipers, average tumour volume pooled per group, error bars represent SD. (**B**) Mice were euthanized after 6 weeks and the tumours removed and photographed (right) and average tumour weight in grams was measured (left). Error bars represent SD. Student *t*-test was performed to calculate statistical significance (* *p* < 0.05, ** *p* < 0.001). (**C**–**J**) RD Flag-Empty and Flag-Tbx3+2a mouse xenograft tumour sections were processed and stained with haematoxylin and eosin (H&E) for histopathological analyses. (**C**–**F**) RD Flag-Empty tumour tissue sections with classic diffuse round polygonal cells, tumour giant cells (C, white arrow), mitotic cells (**D**, red arrows) and invasion into skeletal muscle (**E**,**F**, black arrows). (**G**–**J**) RD Flag-Tbx3+2a tumour tissue sections with predominant fascicular growth of round and spindle-shaped cells (**H**,**J**, white arrows) and invasion into skeletal muscle (**G**,**H**, black arrows) and adipose tissue (**I**,**J**, black arrows). (**K**) Immunohistochemical analysis of RD Flag-Empty and Flag-Tbx3+2a mouse xenograft tumour tissues sections stained with antibodies specific to the EMT markers, Vimentin and β-Catenin. Representative phase contrast light microscopic images were taken at 200× and 400× magnification.

We next investigated the in vivo tumour-forming ability of TBX3 overexpressing cells by injecting RD Flag-Empty and Flag-Tbx3+2a cells subcutaneously into immunocompromised nude mice. The mice injected with Flag-Tbx3+2a cells developed tumours as early as 4 weeks, whereas tumours were only visible after 5 weeks in mice injected with Flag-Empty cells (Figure 3A left panel). Furthermore, the average volume of tumours in the mice injected with Flag-Tbx3+2a cells was larger when measured with callipers (Figure 3A right panel). These observations were consistent with the results from the in vitro assays, which suggested that TBX3 promotes the ERMS phenotype. Surprisingly, however, when the tumours were excised at the 6 week end point, the Flag-Empty tumours were larger and weighed significantly more than the Flag Tbx3+2a tumours (Figure 3B). We speculated that the Tbx3+2a-expressing tumour cells have enhanced migratory/invasive ability, which enabled them to leave the primary tumours as single cells and/or clusters of cells [73]. To test this, we performed histopathological analyses and our results show that all tumour sections stained with haematoxylin and eosin (H&E) displayed general pleomorphic morphology, with cells differing in shape and size. Importantly, while Flag-Empty tumour tissue sections displayed cells with a classic diffuse round polygonal appearance (Figure 3C–F), Flag-Tbx3+2a tumour cells showed predominant fascicular cell growth, in which round and spindle-shaped cells appear to grow in cords of differing polarities, suggestive of a motile phenotype (Figure 3G–J). Indeed, immunohistochemical analyses show that the Flag-Tbx3+2a cells had a more invasive phenotype because they stained more intensely for the EMT markers, Vimentin and β-Catenin (Figure 3K).

### 2.5. TBX3 Knockdown Inhibits Cell Proliferation, Induces Apoptosis and Blocks Anchorage Independent Growth and Migration of ERMS Cells 

**Figure 4:**
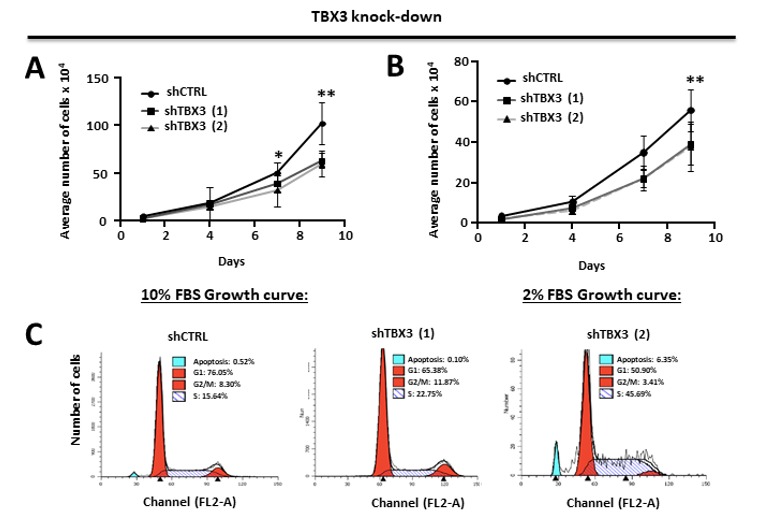

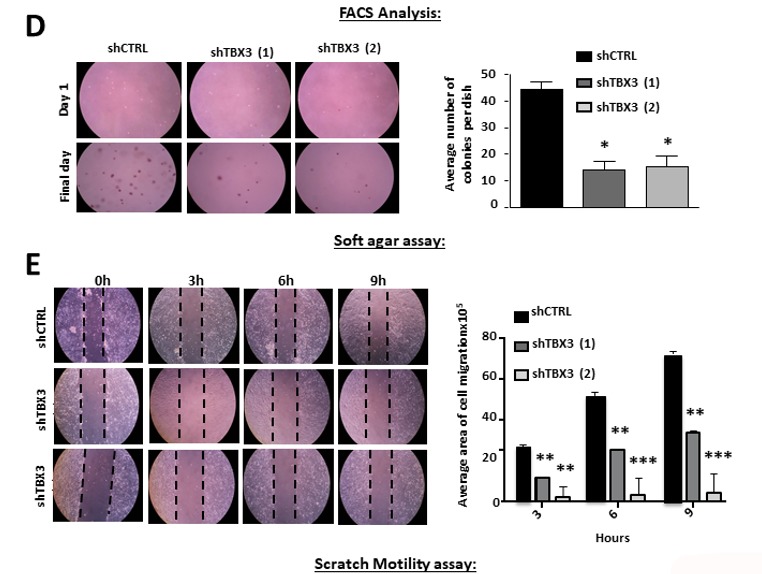
TBX3 knock-down inhibits cell proliferation, induces apoptosis and blocks anchorage-independent growth and migration of ERMS cells. (**A**,**B**) Growth curve analysis of RD shCTRL and shTBX3 cell lines were performed in media supplemented with either (**A**) 10% fetal bovine serum (FBS) or (**B**) low serum conditions (2% FBS). (**C**) Fluorescence-assisted cell sorting of indicated cell lines. Percentages of cells in each phase of the cell cycle are indicated. (**D**) RD shCTRL and shTBX3 cell lines were suspended in soft-agar medium slurry and allowed to proliferate for 30 days. Whole dishes were stained with p-iodinitrotetrazolium, and representative images are shown (200X magnification). (Left) Representative images are shown (200× magnification). (Right) Quantification of at least 40 fields of view from three independent experiments. (**E**) Cell motility assays were performed to measure the migration of RD shCTRL and shTBX3 cells. A linear wound was made on confluent cells and distance migrated was measured at 3, 6 and 9 h. The values in the graphs represent the mean of at least four independent experiments ± SEM (* *p* < 0.05, ** *p* < 0.001, *** *p* < 0.0001).

The observations above provided evidence that TBX3 overexpression promotes several oncogenic processes and that TBX3 may be a druggable target for the treatment of ERMS. To validate this biologically, we next investigated if depleting TBX3 by shRNA in RD cells could inhibit the cancer phenotype. The results show that, compared to shCTRL cells, the shTBX3 cells proliferated significantly slower over nine days under standard (10% serum) culture conditions (Figure 4A) and low serum (2%) conditions (Figure 4B). This suggests that the growth of RD cells is proportional to the levels of growth factors in their environment and that depleting TBX3 significantly decreases cell proliferation, but that it does not contribute to growth factor independence. 

The next set of experiments therefore determined the cell cycle profile of shCTRL and shTBX3 cells cultured in standard 10% FBS using fluorescence assisted cell sorting (FACS). The results show that the shCTRL and shTBX3 (1) cells exhibited a profile typical of dividing cells (Figure 4C). Interestingly, the shTBX3 (2) cell line displayed an increased percentage of apoptotic cells, which provides support for previous studies showing that TBX3 promotes cancer cell survival through its ability to evade apoptosis [24]. In addition, the shTBX3 (2) cell line also had an increase percentage of cells in S-phase, which is consistent with a previous report that TBX3 may be required for cells to transit through S-phase and that this function may be linked to its role as a pro-proliferative factor [50]. It is worth noting that, compared to the shTBX3 (1) cell line, the levels of TBX3 were always lower in the shTBX3 (2) cell line (see Figure 1C) and that these cells proliferated slower, albeit not statistically significant. This suggests that the impact of TBX3 on apoptosis and the cell cycle may be dependent on intracellular levels of TBX3.

Furthermore, our results show that in soft agar and scratch motility assays, shTBX3 cells formed significantly fewer colonies (Figure 4D) and migrated significantly slower (Figure 4E) compared to the shCTRL cells. Taken together, our data suggest that TBX3 is a powerful oncoprotein in ERMS and that it may be a novel target for therapeutic interventions to treat this cancer.

### 2.6. c-Myc Transcriptionally Upregulates TBX3 in ERMS

**Figure 5:**
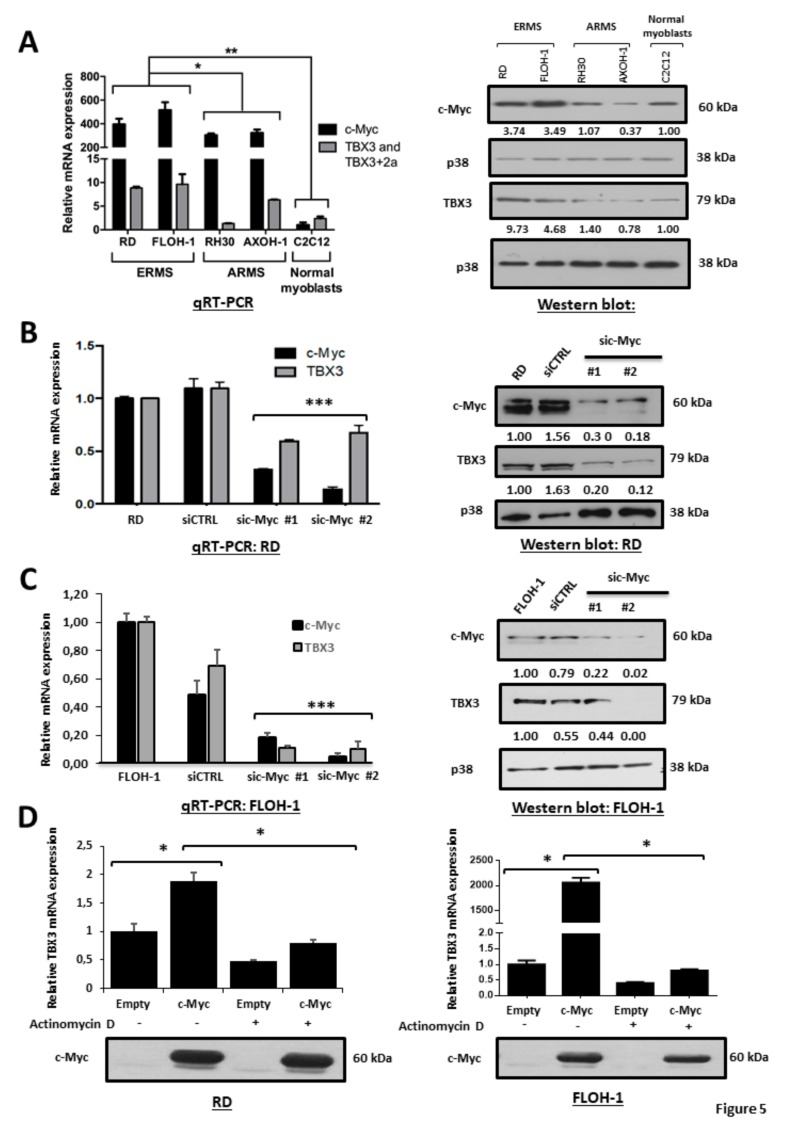
c-Myc transcriptionally upregulates TBX3 in ERMS. (**A**) Total RNA and protein were extracted from the indicated RMS cell lines and normal muscle myoblasts (C2C12) and (left) qRT-PCR was performed on reverse transcribed RNA using primers specific to c-Myc, TBX3, TBX3+2a and either GUSB or β-Actin (housekeeping genes). mRNA levels for c-MYC were normalised to β-Actin and mRNA levels for the two TBX3 isoforms were normalised to GUSB and the results were quantified relative to the results obtained for C2C12. (Right) protein extracts (the same used in Figure 1A) were subjected to Western blot analysis with antibodies specific to c-Myc, TBX3 (same blot as shown in Figure 1A) and p38 (loading control). (**B**) RD and (**C**) FLOH-1 cells were transiently transfected with 50 nM siRNAs to c-Myc (sic-Myc#1 and sic-Myc#2) or control siRNA (siCTRL) for 48 h. Extracts were subjected to (left) qRT-PCR and (right) Western blot analyses using primers or antibodies to c-Myc and TBX3 respectively. Densitometric readings for all Western blots were calculated relative to the p38 control. (**D**) RD and FLOH-1 cells were transfected for 48 h with 500 ng pcDNA-Flag-c-Myc or pcDNA-Flag-Empty expression constructs, then treated with 5 μg/mL Actinomycin D or vehicle (DMSO) for 30 min. (Upper) qRT-PCR was performed on reverse-transcribed RNA using primers to TBX3 and mRNA levels were normalized to β-Actin. The values in the graphs represent the mean of three independent experiments ± SEM (* *p* < 0.05; ** *p* < 0.001; *** *p* < 0.001). (Lower) protein extracts were subjected to Western blot analysis with an antibody specific to c-Myc.

To begin to explore the mechanism(s) by which TBX3 is upregulated in ERMS, we speculated that c-Myc may be involved. The rationale for this was that c-Myc is overexpressed in RMS tumours and cell lines [49,74] and it is important for driving and sustaining rhabdomyosarcomagenesis [38,42,43,48]. Moreover, c-Myc upregulates TBX3 transcriptionally in chondrosarcoma cells through two E-box motifs [50]. We firstly investigated whether there was a correlation between c-Myc and TBX3 mRNA and protein levels in ERMS and ARMS cell lines. Our results show that, compared to normal myoblasts and ARMS cells, the ERMS cell lines had higher levels of c-Myc and TBX3 mRNA and protein (Figure 5A). Furthermore, depleting c-Myc in RD and FLOH-1 cells by siRNA (sic-Myc #1 or sic-Myc #2) resulted in a corresponding decrease in TBX3 mRNA and protein levels, suggesting that c-Myc may be transcriptionally upregulating TBX3 (Figure 5B,C**)**. To confirm this, c-Myc was ectopically overexpressed in ERMS cells in the presence or absence of Actinomycin D, an inhibitor of de novo transcription, and TBX3 mRNA levels were measured. Our results show that the inhibition of transcription abolished c-Myc-mediated activation of TBX3 expression (Figure 5D). 

### 2.7. AKT1 Is the Predominant AKT Isoform in ERMS and Post-Translationally Upregulates TBX3 

**Figure 6:**
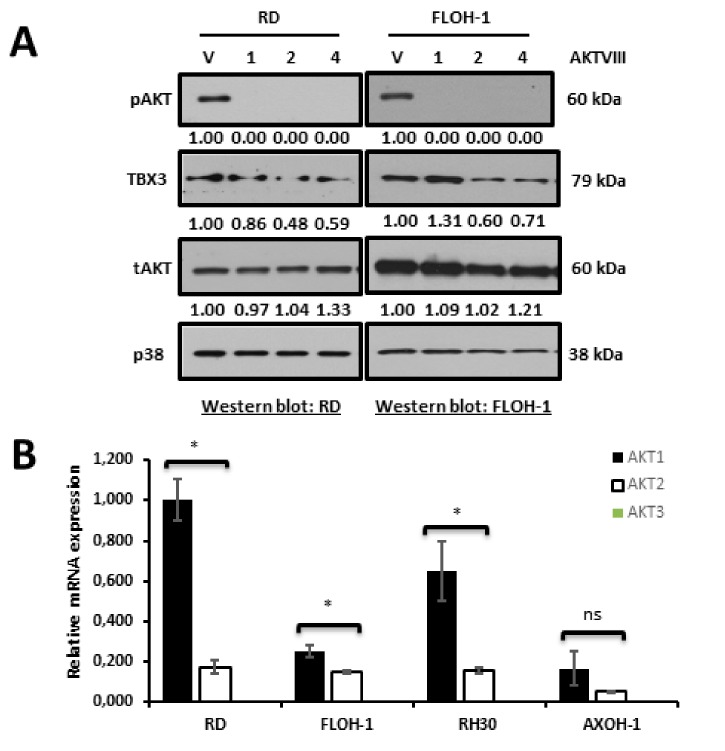

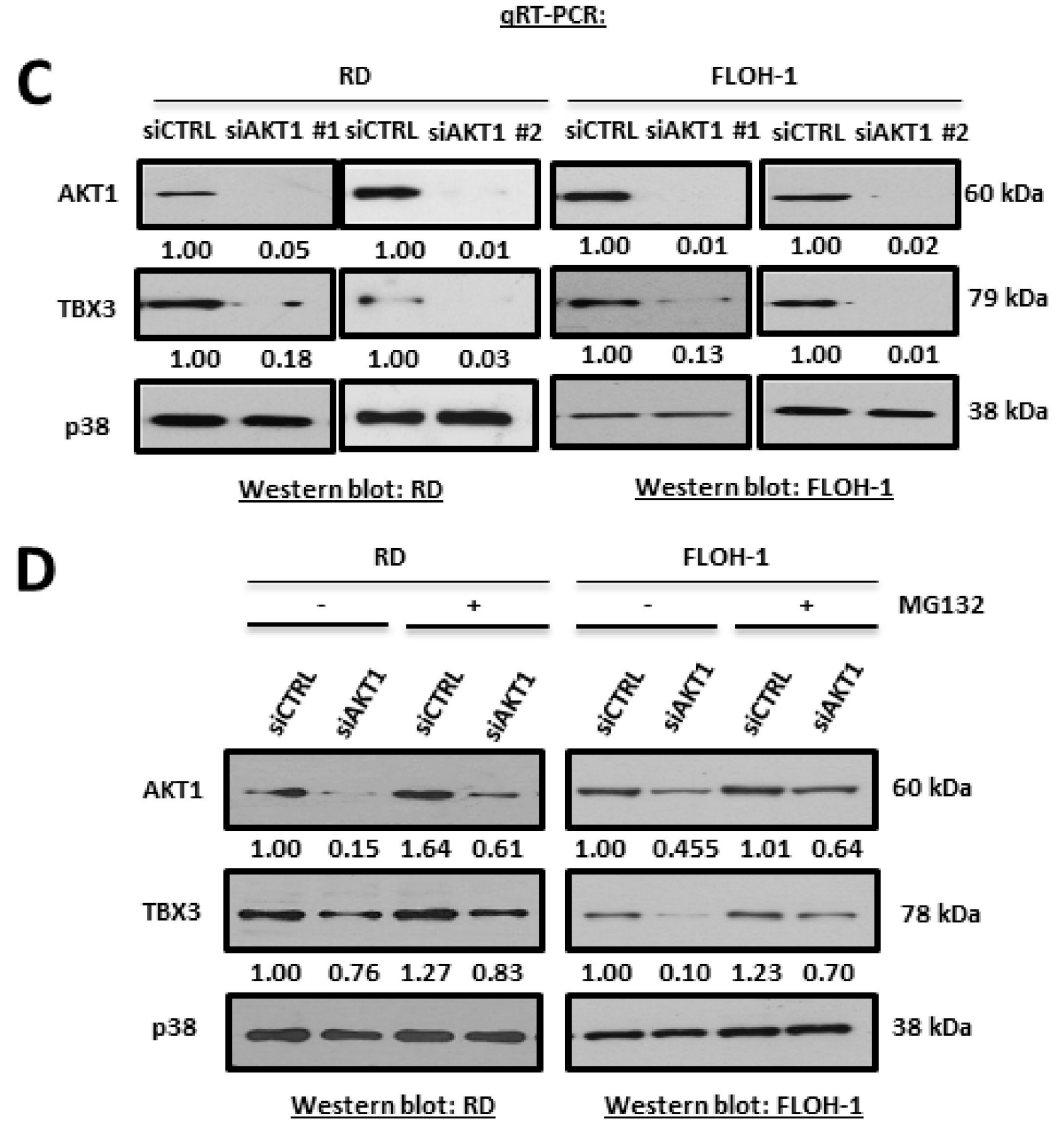
AKT1 is the predominant AKT isoform in ERMS and post-translationally upregulates TBX3 in ERMS. (**A**) RD (left) and FLOH-1 (right) cells were treated with 10 μM of AKT inhibitor (AKT VIII) or vehicle (V; DMSO) for 1, 2 and 4 h. Protein extracts were subjected to Western blot analyses with the indicated antibodies. (**B**) qRT-PCR of cDNA extracted from indicated RMS cell lines cells using primers specific to AKT1, AKT2 and AKT3 and β-Actin (housekeeping gene) and mRNA levels were normalised to β-Actin and quantified relative to the RD cell line. The values in the graph represent the mean of at least three independent experiments ± SEM (* *p* < 0.05; ns, not significant). (**C**) RD (left)and FLOH-1 (right) cells were transiently transfected with 50 nM siAKT1 #1 for both cell lines or 25 nM and 75 nM siAKT1 #2 for RD and FLOH-1 cells, respectively, or the equivalent concentrations of siCTRL for 48 h. Protein extracts were subjected to Western blot analyses with the indicated antibodies. (**D**) RD and FLOH-1 cells were transfected with siCTRL and siAKT1 for 42 h then treated with the proteasome inhibitor 20 µM MG132 for 30 min, protein was extracted and subjected to Western blotting using the indicated antibodies. Densitometric readings for all Western blots were calculated relative to the p38 control.

Rhabdomyosarcomagenesis involves, in part, the prolonged activation of serine/threonine kinases such as AKT [60], and we have previously shown that AKT1 and AKT3 phosphorylation stabilizes the TBX3 protein in chondrosarcoma/fibrosarcoma and melanoma, respectively [50,71]. We therefore determined whether the AKT-signalling pathway may also be involved in upregulating TBX3 in ERMS. To this end, RD and FLOH-1 cells were treated with the AKTVIII inhibitor and the levels of TBX3 measured by Western blotting. Our results show that inhibiting the AKT pathway resulted in a decrease in pAKT at all the timepoints tested and a reduction in TBX3 protein levels at 2 and 4 h (Figure 6A). There are three AKT isoforms, namely AKT1, AKT2 and AKT3, and we therefore wished to determine which isoform(s) was responsible for regulating TBX3 levels in ERMS. To this end, qRT-PCR analyses were performed to determine which of the AKT isoforms was most abundantly expressed in ERMS. Our data reveal that AKT3 was not expressed in any of the RMS cell lines tested and that AKT1 was the predominant isoform in these cells (Figure 6B). Importantly, depleting AKT1 by siRNA resulted in a substantial decrease in TBX3 protein levels (Figure 6C). Furthermore, treatment with MG132, an inhibitor of the proteasome, rescued TBX3 levels in ERMS cells transfected with siAKT1 (Figure 6D). These results confirm that AKT1 regulates TBX3 post-translationally.

## 3. Discussion

Embryonal rhabdomyosarcoma (ERMS) accounts for 75% of all RMS cases and occurs mostly in children [2,4]. Despite newer and more intensive chemotherapeutics, the prognosis for high-risk, recurrent and metastatic ERMS remains poor (10–30% five-year survival rate) [6,9]. There is, therefore, still a need to discover more effective treatments that will reduce rates of relapse and improve clinical outcomes for ERMS. In this regard, targeted therapies have gained significant traction in cancer treatment but very few effective drug targets have been identified in RMS. To address this requires the identification of key transcription factors, cell cycle regulators and mitogenic signalling molecules/pathways that are altered in RMS. Here, we reveal a novel c-Myc/AKT1/TBX3 axis that plays an essential role in maintaining the ERMS phenotype. 

The overexpression of TBX3 is a key feature of several cancers, where it promotes several oncogenic processes, but little is known about its expression status and function in RMS [20,24,28,31]. Our results show that TBX3 is expressed in ERMS and ARMS but that it is expressed at higher levels in ERMS. These data are consistent with microarray and RNA-seq data, from the NIH Paediatric Oncology Branch Oncogenomics and the Cancer Cell Line Encyclopedia (CCLE) databases, respectively, which show that TBX3 is expressed at higher levels in ERMS compared to ARMS and skeletal muscle cells [36,75,76]. Together, this suggests that TBX3 may play an important role in contributing to the ERMS phenotype. Indeed, here we show, using ERMS cell culture models in which TBX3 was either stably overexpressed or knocked down, that it promotes cell proliferation, anchorage-independent growth, tumour formation and cell migration and invasion. These data provide compelling evidence that TBX3 is a key oncogenic transcription factor in ERMS and that it may be a novel drug target to treat this childhood cancer. Furthermore, we show that c-Myc and AKT1, transcriptionally and post-translationally, respectively, upregulate TBX3 in ERMS. These results are significant because c-Myc and AKT are well-established drivers of ERMS, and they therefore suggest that TBX3 lies downstream of important oncogenic pathways in this sarcoma.

Results from our study are exciting because, while historically considered undruggable, transcription factor activity has been successfully targeted preclinically and clinically using multiple approaches. These include inhibiting transcription factor-DNA binding, disrupting transcription factor–protein co-factor interactions, and altering transcription factor levels by inhibiting upstream regulators or modulating ubiquitylation and subsequent proteasome degradation [77]. For example, Omomyc is an MYC-dominant negative gene product and was the first Myc-specific inhibitor to reach clinical trials. Omomyc was shown to inhibit c-Myc transcriptional activity by disrupting its interaction with Max, and therefore its ability to bind and activate its target genes [78,79,80]. In addition, the PI3K/AKT/mTOR pathway upregulates c-Myc levels and inhibiting either mTORC1 by Rapamycin/RAD001/CCl-779 or AKT with MK2206 has been shown to inhibit c-Myc levels. This was associated with great therapeutic efficacy in various cancers including acute myeloid leukaemia (AML), T-cell lymphoblastic leukaemia, multiple myeloma, breast cancer and non-small lung cancer [81,82,83,84,85,86]. Furthermore, transcription of *c-Myc*, controlled by the epigenetic regulator, BRD4, and inhibition of BRD4 by the small molecule inhibitor, JQ1, showed potent anti-cancer activity in vitro and in vivo of several cancers, for example, multiple myeloma, AML and Burkitt’s lymphoma [87,88,89,90]. A newly developed technology termed proteolysis targeting chimaera (PROTAC) was shown to have efficacy superior to small molecules in targeting transcription factors. This approach utilizes the ubiquitin–protease system to target a specific protein and induces its ubiquitylation and subsequent degradation [91]. For example, ARV-825, a PROTAC designed to specifically target BRD4, was more effective than JQ1 at depleting levels of c-Myc and its downstream targets [92]. Taken together, these studies reveal successful approaches for inhibiting the oncogenic activity of transcription factors such as TBX3. 

Single targeted approaches have not shown sustained activity for the treatment of ERMS and other cancers. Indeed, popular targeted therapies for RMS include mTOR and Hedgehog inhibitors and RTK-specific monoclonal antibodies or small-molecule inhibitors but, while an initial response is observed with these treatments, the disease often continues to progress and/or tumour drug-resistance develops. In light of our data that show that c-Myc and AKT1 transcriptionally and post-translationally, respectively, upregulate TBX3 in ERMS, it would be interesting to explore ways of inhibiting all three of these oncogenic proteins. In this regard, it is worth noting that BAY 1125976, a selective allosteric AKT1/2inhibitor, exhibits high efficacy in AKT signalling-dependent breast, prostate and anal tumour growth in mouse models [93]. It would therefore be interesting to determine whether Omomyc, ARV-825, MK2206 and BAY 1125976 are able to decrease TBX3 levels in ERMS, and whether administering them together with an siTBX3 functions synergistically to inhibit the ERMS phenotype. 

In conclusion, our results reveal that c-Myc/AKT1/TBX3 is an important axis that maintains the ERMS phenotype and identifies multiple targets that can be used in combination for the treatment of ERMS.

## 4. Materials and Methods 

### 4.1. Cell Culture and Transfections

AX-OH-1 human ARMS and FL-OH-1 human ERMS cells (kindly provided by Professor Stefan Bath, University of Cape Town) were cultured in Roswell Park Memorial Institute Medium (RPMI)-1640 (Sigma-Aldrich, Darmstadt, Germany). RH30 human ARMS cells (kindly provided by Associate Professor Judith Davie, Southern Illinois University), RD human ERMS cells (ATCC^®^ CCL-136™) and C2C12 mouse myoblast cells (ATCC^®^ CRL-1772™) were cultured in Dulbecco’s Modified Eagle’s medium (DMEM) (Sigma-Aldrich, Germany). Media was supplemented with heat-inactivated 10% fetal bovine serum (FBS, Gibco, Gaithersburg, MD, USA), 100 U/mL penicillin and 100 μg/mL streptomycin. Cells were maintained at 37 °C in an atmosphere of 5% CO_2_ and 65% humidity. 

Transient knock-down of c-Myc and AKT1 expression was achieved by transfecting cells with siRNAs that specifically target mRNA expression of c-Myc (sic-Myc) or AKT1 (siAKT1). RD and FLOH-1 cells were plated at either 4 × 10^4^ cells/well of a 24-well plate or 7 × 10^4^/well of a 12-well and transfected with 50 nM sic-Myc #1 (Dharmacon, Lafayette, CO, USA), 50 nM sic-Myc #2 (SI02662611;Qiagen, Germantown, MD, USA), 25 nM siAKT1 #1 (L-003000-00-0005; Dharmacon, USA), 75 nM siAKT1 #2 (AM16708; Ambion-Life technologies, Grand Island, NY, USA) or a control siRNA (non-silencing, siCtrl) (1027310; Qiagen, USA), according to manufacturer’s instructions. Briefly, siRNAs were diluted in 97 μL serum-free medium, after which 3 μL of HiPerFect^®^ (Qiagen, USA) was added directly to the siRNA solution, incubated at room temperature for 15 min, and added to cells. 

Overexpression of c-Myc was achieved as follows: RD and FLOH-1 cells were plated at 2 × 10^5^ and 1.5 × 10^5^ cells per well, respectively, in 6-well plates and, the next day, transiently transfected with 500 nM pCDNA-Flag c-Myc (kindly provided by Professor Luscher from Institut für Biochemie, Planegg, Germany) or pCDNA empty vector using XtremeGENE HP DNA transfection reagent (Roche, Basel, Switzerland), according to the manufacturer’s instructions. Briefly, DNA was diluted in 100 µL of DMEM, and 3 µL of XtremeGENE HP DNA transfection reagent (Roche, Switzerland) was then added and incubated at room temperature for 10 min. The transfection mixture was added dropwise to the cells and incubated for 48 h.

### 4.2. Generation of Stable Cell Lines 

To generate cell lines in which TBX3 mRNA levels were knocked down, RD cells were stably transfected with the pSuper.neo/GFP expression vector containing a sequence targeted to TBX3 (shTBX3), or a nonspecific control (shCTRL), as previously described [32]. Stable transfectants were selected in growth medium containing 800 μg/mL G-418 (Promega, San Luis Obispo, CA, USA). Effective TBX3 knockdown was assessed by Western blot analysis. Tbx3-overexpressing cell lines were generated by stably transfecting RD cells with a Flag-tagged pCMV-empty vector, pCMV-Tbx3 and/or pCMV-Tbx3+2a vector (kindly provided by Professor Colin Goding at the Ludwig Institute of Cancer Research, Oxford, UK) using FuGENE^®^HD (Roche Molecular Biochemicals, Mannheim, Germany) according to the manufacturer’s instructions. Stable transfectants were selected for with 800 μg/mL G-418 antibiotic (Promega, USA). Effective overexpression of Flag-Tbx3 and Flag-Tbx3+2a was assessed by Western blot analysis with an antibody to FLAG. 

### 4.3. Western Blot Analysis 

Cells were harvested and protein prepared as described previously [94]. Primary antibodies used were as follows: rabbit polyclonal anti-TBX3 (1:500; 42-4800; Zymed, South San Francisco, CA, USA) or rabbit polyclonal anti-TBX3 (1:1000; AB99302 Abcam, Cambridge, MA, USA), mouse monoclonal anti-Flag M2 (1:1000; F1804; Sigma-Aldrich, Germany), rabbit polyclonal anti-c-Myc (1:1000); sc-N262; Santa Cruz Biotechnology, Santa Cruz, CA, USA), rabbit polyclonal AKT (1:1000;9272S; Cell Signalling, Danvers, MA, USA), rabbit polyclonal p-AKT (1:1000; 9271S ; Cell Signalling, USA), rabbit polyclonal AKT1 (1:1000) (C73H10 ; Cell Signalling, USA) and rabbit polyclonal anti-p38 (1:5000; M0800, Sigma Aldrich, Germany). Secondary antibodies, horseradish peroxidase (HRP)-conjugated goat anti-mouse (Biorad, Hercules, CA, USA), goat anti-rabbit (Biorad, USA) or donkey anti-goat (Santa Cruz Biotechnology, Santa Cruz, CA, USA) were used at a 1:5000 dilution. Immunoblots were detected with enhanced chemiluminescence (ECL) (Pierce, Waltham, MA, USA) as previously described. 

### 4.4. Quantitative Real-Time Polymerase Chain Reaction (qRT-PCR)

Total RNA was extracted from cells using the RNeasy Plus Mini kit (Qiagen, USA) [50]. Reverse transcription of RNA (1μg) was performed according to the manufacturer’s instructions using the InProm-IITM reverse transcription system (A3800; Promega, USA). Using 2 μL of cDNA, quantitative real time PCR was conducted on an Applied Biosystems StepOne Plus thermal cycler or LightCycler Version 3 (Roche, Switzerland) using 2× SYBR green master mix (Applied Biosystems, Beverly, MA, USA), or SensiMix Lite Kit (QT 405-05; Quantace, Carlsbad, CA, USA) respectively, according to the manufacturer’s protocols. PCR cycle parameters were: denaturation for 15 min at 95 °C, combined annealing and extension for 35 cycles at 60 °C for 1 min. Each DNA sample was quantified in triplicate and a negative control without cDNA template was run with every assay to assess the overall reaction specificity. Melting curve analyses were carried out to ensure product specificity. Relative mRNA expression levels were normalized to glucuronidase beta (GUSB/Gusb) or β Actin using the 2-ΔΔCt method. The following primers were used to amplify the human TBX3 (QT00022484, Qiagen, USA), TBX3 (forward primer 5′-CAATTCTCGGTGGATGGTG-3′ and reverse primer 5′-GGCTGGTATTTGTGCATGG-3′ purchased from Integrated DNA Technologies, Coralville, IA, USA), TBX3+2a (forward primer 5′-CAATTCTCGGTGGATGGTG-3′ and reverse primer 5′-GTAGCGTGATCACTTGGGA-3′ purchased from Integrated DNA Technologies, USA), c-Myc (forward primer 5′ CTGAGACAGATCAGCAACAACC3′ and reverse primer 5’TTGTGTGTTCGCCTCTTGAC3’ purchased from Integrated DNA Technologies, USA), GUSB (QT00046046; Qiagen, USA), β Actin (forward primer 5’CGGCATCGTCACCAACTG3’ and reverse primer 5’AACATG ATCTGGGTCATCTTCTC3’ purchased from Integrated DNA Technologies, USA) and mouse Gusb (forward primer 5′-ACTGACACCTCCATGTATCCCAAG-3′ and reverse primer 5′-CAGTAGGTCACCAGCCCGATG-3′ purchased from Integrated DNA Technologies, USA).

### 4.5. Proliferation Assay 

Short-term growth of the TBX3 knock-down and overexpression cell lines was performed in DMEM supplemented with 10% or 2% (v/v) FBS. 1 × 10^4^ RD cells were plated per well in triplicate in 12-well plates. Growth curves were performed over a seven or nine day period, as previously described [94]. 

### 4.6. Anchorage Independence Assay

Soft agar assays were performed in 35 mm dishes, which were layered first with 0.5% agar (Sigma Aldrich, Germany) in cell culture medium, followed by 0.35% agar in cell culture medium containing 5000 cells. Colonies were stained with p-iodonitrotetrazolium chloride (1 mg/mL, Sigma Aldrich, Germany), incubated overnight at 37 °C, and photographed. 

### 4.7. In Vitro Cell Migration Assay

Cell migration was measured using a two-dimensional in vitro scratch motility assay as previously described [32]. Briefly, cells were grown in 24-well plates to 100% confluency, after which an artificial wound was introduced in the center using a sterile pipette tip. The distance migrated was measured over time (3, 6 and 9 h) and calculated using ImageJ software (National Institute of Health, National Institute of Health, MD, USA). 

### 4.8. Xenograft Mouse Model

All protocols employed in this study were approved by and performed in accordance with the University of Cape Town Animal Research Ethics Committee (015/027) guidelines for the care and use of laboratory animals [36]. Tumorigenicity experiments were performed by subcutaneously injecting 1 × 10^7^ RD Flag Empty or Flag-Tbx3 cells in 100 μL PBS into the right flanks of 4 to 6-week-old MF-1 nude mice. In situ tumour growth was measured using calipers and the formula [Volume mm3 = (length) × (width^2^) × 0.5]. At the 6 week endpoint of the study, the mice were euthanised and organs, including tumours, were removed for histopathological analyses. Samples were processed in an automatic tissue processor (Shandon Duplex) for routine paraffin wax embedding. Tissue sections were cut, mounted onto glass slides, stained with haematoxylin and eosin and viewed using a light microscope (Zeiss, Cologne, Germany).

### 4.9. Immunohistochemistry

Paraffin-embedded mouse xenograft tumour sections were cut at 4 µm, deparaffinized in xylenes and rehydrated through a graded series of alcohols. Antigen retrieval was achieved by incubation in a target-retrieval solution from the EnVision FLEX Mini Kit, High pH (Link) (Dako, Carpinteria, CA, USA) as per manufacturer’s instructions. All subsequent steps were carried out at room temperature. The tissue sections were blocked firstly in peroxidase-blocking buffer-blocking solution (K8000; Dako, USA) for 5 min, followed by 1 h in 2.5%BSA/TBS to prevent non-specific binding. Sections were then incubated overnight at 4 °C with mouse monoclonal anti-Flag M2 (1:1000; F1804; Sigma-Aldrich, Germany), rabbit monoclonal anti-β-Catenin (1:25; D10A8; Cell Signalling, USA) and rabbit polyclonal anti-Vimentin (1:100; R28; Cell Signalling, USA), followed by incubation for 1 h with an HRP secondary antibody (K400211; Dako, USA). Sections were subsequently incubated for 10 min in Elusion substrate buffer containing DAB chromogen solution (K346711; Dako, USA) for colour development. All sections were counterstained with Mayer’s Haematoxylin and Scott’s solution, dehydrated and cleared with xylenes, and mounted using Entellan (107960; Merck, Darmstadt, Germany). Sections that were not incubated with the appropriate primary antibody were included as negative controls. All imaging was performed using the Zeiss bright field microscope.

### 4.10. Fluorescence Assisted Cell Sorting

The cell cycle phase distribution was assessed by measuring DNA content by flow cytometry as described previously [95]. Briefly, cells were collected by trypsinisation, washed twice with 1× PBS, resuspended in 2 mL of cold PBS and counted on a haemocytometer to determine the volume of propidium iodide (PI) solution (2 mM MgCl_2_, 10 mM Pipes buffer, 0.1 M NaCl, 0.1% Triton X-100, 0.01 mg/mL PI) that would later be added. Cells were fixed in 8 mL of 70% cold ethanol for at least 30 min at −20 °C. Fixed cells were collected by centrifugation at 1500 rpm for 5 min at RT, washed twice with 1× PBS and centrifuged at 6000 rpm for 1 min at RT. Before flow cytometry analyses, the samples were treated with RNase A (50 μg/mL) for 15 min at 37°C and immediately stained for 30 min at RT with PI solution, to yield a final concentration of 1 × 10^6^ cells/mL. A minimum of 50,000 cells/sample were subjected to analysis using a Beckman Coulter FACS Calibur flow cytometer (Beckman Coulter, Miami, FL, USA). Cell cycle profiles were analysed using the Modfit LTTM (Verity Software House, Topsham, ME, USA) Software.

### 4.11. Treatments 

To inhibit the kinase activity of the AKT1, 2 and 3 isoforms in RD and FLOH-1 cells, 7 × 10^4^ cells were plated in a 12-well plate and, when 60–70% confluent, serum starved in 0.5 % FBS for 24 h, after which cells were treated with 10 μM of AKT-VIII chemical inhibitor (124018, Calbiochem, San Diego, CA, USA) for 1, 2 and 4 h, followed by protein harvest and Western blot analyses. For the inhibition of de novo transcription, RD and FLOH-1 cells were treated with vehicle only (DMSO) or 5 µg/mL Actinomycin D (Sigma, St. Louis, MO, USA) for 30 min in the dark. After this, cells were harvested and protein lysates used for Western blotting. For the inhibition of ubiquitin-mediated protein degradation, RD and FLOH-1 cells were treated with 20 µM MG132 (Calbiochem, USA) for 30 min following siRNA transfections.

### 4.12. Statistical Analysis

Statistical significance was determined using the student’s *t*-test (Excel, Microsoft, Microsoft, Redmond, WA, USA). Significance was accepted at *p* < 0.05.

## 5. Conclusions

In conclusion, this study shows that TBX3 promotes substrate-dependent and -independent proliferation, migration and tumour formation of ERMS. We further reveal that TBX3 is upregulated by c-Myc transcriptionally and AKT1 post-translationally. The c-Myc/AKT1/TBX3 pathway is identified as an important axis to target for more effective treatment of ERMS.

## Supplementary Materials

Supplementary File 1
